# Anorexia Nervosa: Evaluating Disparities in Places of Death in the United States Over 22 Years Using the CDC WONDER Database

**DOI:** 10.7759/cureus.51245

**Published:** 2023-12-28

**Authors:** Nirmal Patel, Rahul Tyagi, Deepanwita Biswas, Ayesha Birjees, Chetana Rajesh, Sadia Khan

**Affiliations:** 1 Internal Medicine, St. George’s University School of Medicine, West Indies, GRD; 2 Family Medicine, Leeds General Practitioner Confederation, Leeds, GBR; 3 Family Medicine, Royal College of General Practitioners, London, GBR; 4 Medicine, Bharati Vidyapeeth's Medical College, Pune, IND; 5 Internal Medicine, Fathima Institute of Medical Sciences, Kadapa, IND; 6 Pediatrics and Child Health, Sri Ramachandra Medical College and Research Institute, Chennai, IND; 7 Internal Medicine, Karachi University, Karachi, PAK

**Keywords:** palliative care, end-of-life care, hospice care, home care, anorexia nervosa, cdc-wonder database, mortality trends

## Abstract

Introduction: Anorexia nervosa is a severe and occasionally fatal eating disorder characterized by extreme weight loss and a distorted body image in which the affected individuals typically exhibit a strong fear of gaining weight, leading to rigid dietary restrictions and excessive activity. This condition can cause severe health problems, such as hunger, cardiovascular issues, and organ destruction. Anorexia nervosa is a key subject for research in the context of end-of-life care disparities due to its psychological and physical challenges.

Aims: This study examines differences in the places of death for people with anorexia nervosa during a 22-year period in the USA, taking into account four important factors: age group, gender, race, and U.S. census region.

Methodology: Data were collected from the CDC WONDER website on August 31, 2023, and spans years 1999 to 2020, using the particular ICD-11 code F50.0 for anorexia nervosa. The study aims to uncover the important determinants impacting the location of death within this specific population using sophisticated statistical methods, including univariate logistic regression.

Results: The analysis of aggregate data yielded notable findings. The patient's principal site of death was at home or in hospice care. Other sites were less prevalent, with medical facilities or nursing homes ranking second. The place of death was highly influenced by age groups with diverse patterns. Gender had no significant impact; however, geographical inequalities were noticeable. Individuals in the Northeast, Midwest, and South were less likely than those in the West to die at home or in hospice care. The location of death was unaffected by race.

Conclusions: In conclusion, this study found that death in home and hospice was more common than in medical or hospital nursing facilities in all four analyzed groups. These findings highlight the critical need for significant advancements in end-of-life care, particularly in home and hospice settings.

## Introduction

Death is a part of life that affects the whole family, not just the person who dies [1]. The lack of end-of-life care results in individuals dying isolated in hospitals and other care facilities. Symptomatic management must be aided by an interdisciplinary team with an integrated approach to enhance the quality of life for people who are dying [2]. Currently, there are about 20 million people worldwide requiring end-of-life care, which makes it a global public health concern [3]. As physicians, it is our goal to bridge the gap in the inaccessibility of end-of-life care so that the entire process is much better for all those who are involved.

The concept of a good death can be completely subjective and complex based on social, cultural, religious, economic, environmental, and medical factors. Patients and their families often regard death in a hospice or at home better than in the hospital or nursing home [4]. Places of death can be at home, hospice, a nursing home, or an inpatient palliative care unit. Death at home means that the patient can have their preferred palliative care and a familiar caregiver. In a nursing home, they can have palliative care and the availability of advance directives [5].

Anorexia nervosa is a psychiatric disorder that is associated with multi-system medical complications and is known to possess the highest mortality rate among all mental health disorders [6]. According to the current guidelines of the Diagnostic and Statistical Manual of Mental Disorders, Fifth Edition (DSM-5), anorexia nervosa is characterized by significant weight loss, constant fear of weight gain, and a lack of acceptance of their low body weight. Amenorrhea is no longer a criterion for anorexia nervosa, as it excludes younger children, prepubertal girls, and postmenopausal women.

In an article by Zipfel et al., risk factors for anorexia nervosa include genetic predisposition, psychosocial status, and individual factors that can cause onset and produce changes in serotonergic neuronal pathways leading to the presentation of the illness [7].

The crucial cause of mortality in anorexia nervosa is a disparity in energy intake and requirements, which causes a hypometabolic condition. Refeeding syndrome is a major complication of anorexia nervosa that is associated with mortality. It occurs due to re-feeding in improper amounts to a long-standing malnourished individual with a down-regulated metabolism. Anorexia nervosa causes many cardiac complications, such as arrhythmia, prolonged QT interval, myocardial atrophy, pericardial effusion, and annular changes, leading to mitral valve prolapse [8].

With the progression of anorexia nervosa, there is trilinear hypoplasia, resulting in leukopenia, anemia, and thrombocytopenia. This occurs due to the replacement of bone marrow fat by mucopolysaccharides. Patients with anorexia nervosa also present with several gastrointestinal complications, such as delayed gastric emptying and acute gastric dilation, which can result in gastric perforation. Gastric perforation can also occur due to superior mesenteric artery syndrome, which is caused by constriction of the third part of the duodenum by the superior mesenteric artery due to atrophy of the fat pad between them. Frequently, there may be elevated aspartate aminotransferase and alanine aminotransferase. This finding of deranged liver enzymes corresponds to hypoglycemia, hypophosphatemia, and a very low body mass index. There are multiple endocrine derangements, such as hypogonadism, due to suppression of the hypothalamic-pituitary axis, which commonly results in amenorrhea in female patients. There may be neurological manifestations such as brain atrophy and permanent impairment of neurocognitive capacity [9].

The aim of this study is to evaluate the disparities in places of death of anorexia patients, based on age, gender, region, and race in the USA.

## Materials and methods

This is a cross-sectional observational study about disparities in place of death due to anorexia nervosa in the USA. The data were extracted on a single day (i.e., August 31, 2023) from the Centers for Disease Control and Prevention Wide-ranging Online Data for Epidemiologic Research (CDC WONDER) (accessed from https://wonder.cdc.gov/), which is a publicly available database that contains a wide variety of public health information.

The underlying cause of death was selected using the International Classification of Diseases-10 (ICD-10) code. The code for anorexia nervosa is F50.0 (anorexia nervosa). The data were collected from year 1999 to 2020. The places of death were divided into three main categories: "home or hospice," "medical or nursing facility," and "others." The parameters assessed were age, gender, census region, and race.

The data were exported into a Microsoft Excel sheet. Total deaths of all years were compared between home or hospice and medical facility or nursing home and analyzed using univariate logistic regression (ULR). ULR is used for calculating the relationship between independent and dependent variables.

## Results

Aggregate data on 1,385 deaths from 1999 to 2020 were obtained for anorexia nervosa from the CDC WONDER database.

Table 1 shows the place of death (i.e., whether it was home, hospice, medical facility, nursing home, or others), by age group, gender, census region, and race.

**Table 1:** The place of death, i.e., whether it was home, hospice, medical facility, nursing home, or others, by age group, gender, census region, and race.

	Home or hospice (n = 670)	Medical facility or nursing (n = 649)	Others (n = 66)
Ten-Year Age Groups			
15-24 years	46	46	10
25-34 years	127	77	13
35-44 years	120	113	11
45-54 years	134	107	16
55-64 years	94	111	0
65-74 years	50	69	0
75-84 years	29	52	0
85+ years	39	45	0
Gender			
Female	616	604	66
Male	54	45	0
Census Region			
Census Region 1: Northeast	117	121	11
Census Region 2: Midwest	122	143	12
Census Region 3: South	174	217	27
Census Region 4: West	245	156	19
Race			
Asian or Pacific Islander	26	0	0
Black or African American	15	0	0
White	626	620	64

By 10-year age groups, we see that the highest number of deaths in home or hospice was 134 in the 45-54-year-old group, and the lowest number of deaths was 29 in the 75-84-year-old group. In medical facilities or nursing homes, the highest number of deaths was 113 in the 35-44-year-old group, and the lowest number of deaths was 45 in the 85+-year-old group. In others, the highest number of deaths was 16 in the 45-54 year group, and zero deaths were reported in all age groups above 55 years. By gender, females had a higher number of deaths than males, with the maximum being 616 female deaths in the home or hospice and the minimum being 0 male deaths in the Others category.

By census region, the highest number of deaths was 245 in homes or hospices in the West, and the lowest number of deaths was 11 in others in the Northeast. By race, the highest number of deaths was 626 for whites in homes or hospices, and the lowest number of deaths was 0 for Asian or Pacific Islanders and black or African Americans in both medical facilities, nursing homes, and others.

Table 2 shows the predictors of home or hospice death for anorexia nervosa. By age, taking 25-34 years as a reference age group, the 25-34 year group was most likely to die at home or hospice with an odds ratio of 1, while the 75-84 year group was least likely to die at home or hospice with an odds ratio of 0.395. By gender, taking females as a reference group, males were more likely to die in a home or hospice with an odds ratio of 1.305, while females were less likely to die in home or hospice with an odds ratio of 1. By region, taking the West as the reference group, the West was most likely to see deaths in home or hospice with an odds ratio of 1, while the South was least likely to see deaths in home or hospice with an odds ratio of 0.509. By race, results are not meaningful since both Asian or Pacific Islanders and Black or African Americans in both medical facilities or nursing homes and others had no deaths.

**Table 2:** The predictors of home or hospice death for anorexia nervosa. The significant p-values (<0.05) are marked with an asterisk(*).

Variables	Univariate Logistic Regression		
	Odds Ratio	95% Confidence Interval	P-value
Age			
15-24 years	0.582	(0.362, 0.935)	0.025*
25-34 years	1.0 (Reference)		
35-44 years	0.686	(0.474, 0.992)	0.045*
45-54 years	0.772	(0.536, 1.112)	0.164
55-64 years	0.6	(0.408, 0.882)	0.009*
65-74 years	0.514	(0.326, 0.808)	0.004*
75-84 years	0.395	(0.233, 0.67)	0.001*
85+ years	0.614	(0.37, 1.019)	0.059
Gender			
Male	1.305	(0.866, 1.968)	0.203
Female	1.0 (Reference)		
Census Region			
Census Region 1: Northeast	0.633	(0.462, 0.868)	0.005*
Census Region 2: Midwest	0.562	(0.414, 0.764)	<0.001
Census Region 3: South	0.509	(0.387, 0.67)	<0.001
Census Region 4: West	1.0 (Reference)		
Race			
Asian or Pacific Islander	17101486.87	(0, Inf)	0.972
Black or African American	17101486.87	(0, Inf)	0.979
White	1.0 (Reference)		

**Figure 1:**
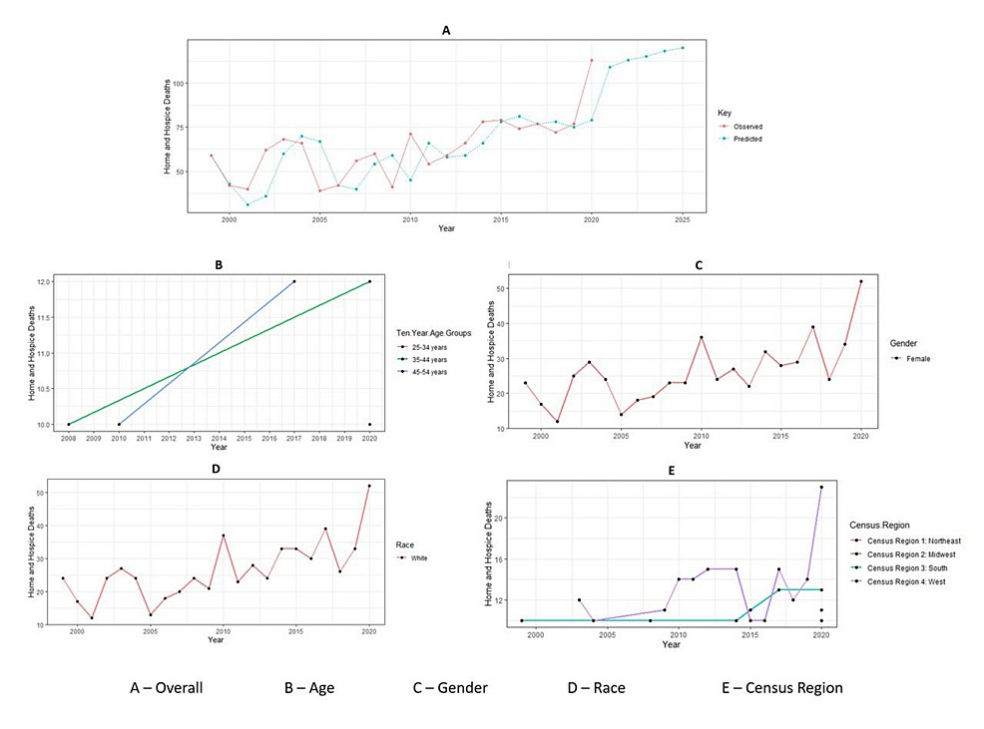
Cumulative home or hospice death trends.

Figure 1 presents the cumulative home or hospice death trends.

## Discussion

To study the mortality trends of anorexia nervosa, 22 years of data between 1999 and 2020 were collected from CDC WONDER. There were a total of 670 home or hospice care patients, 649 patients in medical or nursing facilities, and 66 other patient deaths.

This study yielded several important findings. Based on the age group, the highest number of deaths were in the 45-54 age group for home or hospice care with a total of 134, the 35-44 age group for medical or nursing with a total of 113, and the 45-54 age group for other care with a total of 16. In another study by Stephens et al. on American mortality trends using CDC WONDER on head and neck cancers, 44.5% of patients died in home or hospice care, whereas the remaining 55.5% died in medical or nursing facilities or other places [4]. Moreover, the majority of patients in both locations of death died at the older ages of 55-64 and 65-74. [4]. However, Talha et al. in their study on American mortality trends using CDC WONDER on adult congenital heart diseases reported that 64% of patients were more likely to die at the hospital in the intensive care unit [10]. Younger patients in the age group of 20-34 were more likely to die in hospitals, whereas older patients in the age group of greater than 65 were more likely to die in hospice care [10]. A study on testicular cancer incidence by Gold et al. found that younger (<45), Hispanic, or black men were more likely to die in medical facilities [11]. Moreover, a study by Akinboro et al., on the epidemiological trends associated with the morbidity rate of gonorrhea, found that the morbidity was the highest in the age groups between the ages of 19-24 [12].

Based on gender, females had the highest number of deaths in each category. There were a total of 616 deaths in homes or hospice care, 604 deaths in medical or nursing facilities, and others. In contrast, males (87%) had the highest number of deaths in each category when it came to deaths due to head and neck cancers between 1999 and 2015. This was also similarly seen in the study of mortality trends for adult congenital heart diseases between 2005 and 2018, where a slight majority of deaths (54%) comprised males [10].

This trend holds true as well in the study by Kusnik et al. on mortality trends in colorectal cancer, where men had a higher age-adjusted mortality rate (18.8) compared to women (13.4) [13]. A study by Okobi et al. on mortality trends related to obesity found that, in the age group of 15-24, males made up 60.11% of deaths, and women made up the remaining 39.89% [14].

On the basis of race, whites had the highest number of deaths in each category. There were a total of 626 deaths in homes or hospice care, 620 in medical or nursing facilities, and 64 in other settings. Similarly, Caucasians (92.7%) had the highest number of deaths of the races in the study of mortality trends for head and neck cancers [4]. Caucasians (84%) also made up the highest number of deaths in the mortality trend of adult congenital heart diseases compared to African American and Hispanic populations [10]. According to a retrospective study conducted by Moreno et al., it is also interesting to note that patients with public insurance were half as likely to seek treatment for eating disorders if they were Latin or Asian compared to white patients [15]. In an observational study on cystic fibrosis mortality trends by Singh et al., however, a lower median age of death was observed in Hispanic ethnicities [16].

Based on the census, the highest number of deaths was in Region 4: West for home or hospice care with a total of 245; in Region 3: South for medical or nursing with a total of 217; and in Region 3: South for others with a total of 27. In contrast, patients living in the Southern USA (48.7%) were more likely to die from head and neck cancer in home or hospice care compared to the other census regions, which were closely followed up by the West (48.1%) [4]. A study by Maleki et al. on alcohol-induced mortality trends in 2020 found that there was an increase in mortality in all census groups, but the most notable was the Western Census region [17].

Limitations

The data from the last three years between 2021 and 2023 were not included in the analysis. These missing data would have otherwise been informative about the most recent trends. This would be very important to study in light of major global events, such as the effects of the post-COVID-19 pandemic on the mortality trends of American anorexia patients. Another limitation is that anorexia nervosa was not classified and studied based on its two sub-categories, namely, the restricting type or the binge-eating/purging type.

Future analysis and studies can be done on the basis of data availability through classification based on subcategories.

## Conclusions

From this study, it can be concluded that certain demographic factors, such as age, gender, race, and census region, caused a significant disparity in the mortality trends between different healthcare setups. The first was age, where in the age groups of 15-24, 25-44, 65-74, and 75-84, a significant disparity in mortality trends was seen. A significant disparity across census Region 1 was also noted in healthcare settings when mortality trends were analyzed. Based on these trends, an increase in disparities in the context of significant demographic factors can be predicted with respect to the mortality trends of anorexia. Future measures should be taken to improve end-of-life care for patients.

## References

[1] Eues SK (2007). End-of-life care: improving quality of life at the end of life. Prof Case Manag.

[2] Daly FN, Ramanathan U (2022). End-of-life and hospice care for neurologic illness. Handb Clin Neurol.

[3] Huffman JL, Harmer B (2023). End-of-life care. StatPearls.

[4] Stephens SJ, Chino F, Williamson H, Niedzwiecki D, Chino J, Mowery YM (2020). Evaluating for disparities in place of death for head and neck cancer patients in the United States utilizing the CDC WONDER database. Oral Oncol.

[5] Costa V (2014). The determinants of place of death: an evidence-based analysis. Ont Health Technol Assess Ser.

[6] Gibson D, Workman C, Mehler PS (2019). Medical complications of anorexia nervosa and bulimia nervosa. Psychiatr Clin North Am.

[7] Zipfel S, Giel KE, Bulik CM, Hay P, Schmidt U (2015). Anorexia nervosa: aetiology, assessment, and treatment. Lancet Psychiatry.

[8] Moskowitz L, Weiselberg E (2017). Anorexia nervosa/atypical anorexia nervosa. Curr Probl Pediatr Adolesc Health Care.

[9] Westmoreland P, Krantz MJ, Mehler PS (2016). Medical complications of anorexia nervosa and bulimia. Am J Med.

[10] Talha KM, Kumar P, Ejaz A (2022). Where adults with congenital heart disease die: insights from the CDC-Wonder database. Curr Probl Cardiol.

[11] Gold BO, Ghosh A, Goldberg SI, Chino F, Efstathiou JA, Kamran SC (2023). Disparities in testicular cancer incidence, mortality, and place of death trends from 1999 to 2020: a comprehensive cohort study. Cancer Rep (Hoboken).

[12] Akinboro MK, Mmaduabuchi J, Beeko PK (2023). Epidemiological trends and factors associated with the morbidity rate of gonorrhea: a CDC-Wonder database analysis. Cureus.

[13] Kusnik A, Renjithlal SL, Chodos A, Shanmukhappa SC, Eid MM, Renjith KM, Alweis R (2023). Trends in colorectal cancer mortality in the United States, 1999 - 2020. Gastroenterology Res.

[14] Okobi OE, Beeko PK, Nikravesh E (2023). Trends in obesity-related mortality and racial disparities. Cureus.

[15] Moreno R, Buckelew SM, Accurso EC, Raymond-Flesch M (2023). Disparities in access to eating disorders treatment for publicly-insured youth and youth of color: a retrospective cohort study. J Eat Disord.

[16] Singh H, Jani C, Marshall DC (2023). Cystic fibrosis-related mortality in the United States from 1999 to 2020: an observational analysis of time trends and disparities. Sci Rep.

[17] Maleki N, Yunusa I, Karaye IM (2023). Alcohol-induced mortality in the USA: trends from 1999 to 2020. Int J Ment Health Addict.

